# Tygerberg Research Ubuntu-Inspired Community Engagement Model: Integrating Community Engagement into Genomic Biobanking

**DOI:** 10.1089/bio.2018.0136

**Published:** 2019-12-11

**Authors:** Keymanthri Moodley, Chad Beyer

**Affiliations:** Department of Medicine, Faculty of Medicine and Health Sciences, Centre for Medical Ethics & Law, Stellenbosch University, Cape Town, South Africa.

**Keywords:** biobank, community engagement, genomic, consent

## Abstract

***Introduction:*** Community engagement (CE) is an ethical imperative in research, but the knowledge base for what constitutes effective and ethically sound CE is limited. Ubuntu, as a component of responsive communitarianism where communal welfare is valued together with individual autonomy, is useful in furthering our understanding of effective CE and how it could best be achieved. Similarly, a relative solidarity model serves as a compromise between extreme individualism and extreme communalism and is more appropriate in a heterogenous African context. Approaching CE from an Ubuntu philosophical perspective in southern Africa is particularly important in genomic biobanking, given the implications for individuals, families, and communities.

***Discussion:*** CE is often implemented in a tokenistic manner as an ancillary component of research. Understanding consent information is challenging where genomic biobanking is concerned due to scientific complexity. We started a process of CE around genomic biobanking and conducted empirical research in an attempt to develop a model to promote effective and ethically sound CE, using relative solidarity to create a nuanced application of Ubuntu. The TRUCE model is an eight-step model that uses social mapping to identify potential communities, establishes the scope of CE, and requires that communities are approached early. Co-creation strategies for CE are encouraged and co-ownership of knowledge production is emphasized. Recruiting and engaging communities at each stage of research is necessary. Evaluation and adaptation of CE strategies are included. Discussion and dissemination of results after the research is completed are encouraged.

***Conclusions:*** There is a significant gap between the theory of CE and its authentic application to research in Africa. This Ubuntu-inspired model facilitates bridging that gap and is particularly suited to genomic biobanking. The CE model enhances and complements the consent process and should be integrated into research as a funding and regulatory requirement where applicable.

## Introduction

Community engagement (CE), as a component of Stakeholder Engagement (SE), in biomedical research is increasingly being recognized by researchers and required by some funders as a primary ethical responsibility, with mutually beneficial outcomes for the community and the research team.^1–5^ This importance is emphasized globally by projects such as INVOLVE in the United Kingdom and by PCORI (Patient-Centered Outcomes Research Initiative) in the United States.^6–8^ While generally accepted on principle, the knowledge base for what constitutes effective and ethically sound CE is limited.^9–11^ Furthermore, there is a concern that CE in health research is rarely community directed, often tokenistic in practice, and may not amount to genuine engagement, empowerment, or consideration for the diverse, heterogeneous concerns and priorities of these communities.^1,5,12–18^

The spectrum of engagement can vary from community acknowledgment at a superficial level to community-based action research (CBAR). The focus of this article is on CE, which is a more flexible approach that is not so prescriptive in its intention and outcome. In CBAR, the results of the research are implemented to change the situation being studied,^19,20^ which is beyond the scope of this article.

While CE is acknowledged as being necessary, it is important to start as early as possible and to be nonprescriptive, while ensuring constant evaluation and adaptation of the process, promoting equity in public voices and empowerment of the communities represented.^12,21^ Appropriate CE is likely to build relationships, increase trust, enhance consent processes, and augment the capacity of communities, as occurs in developed country settings. The social contract implemented by UK biobank and the Tissue Trust proposed by Emerson et al. are examples.^22,23^ On the other hand, suboptimal CE is potentially exploitative and disrespectful to communities.^14,24^

Sadly, the latter scenario is often the case in resource-poor settings. Consequently, the principles of CE need to be explicit and include notions such as transparency and respect for the community with genuine reflection on developing authentic research approaches that embrace reciprocity, consensus, and collective decision- making.^25^ CE principles need to be approached and monitored with a strong degree of public oversight to be effective.^26^

In a southern African context, transparent and practical CE approaches can be enhanced by taking into account the concept of Ubuntu.^27,28^ This is particularly important in genetics and genomics research where families and communities are central to the science. Just as the ancient Greeks believed that, *a society grows great when old men plant trees whose shade they know they shall never sit in*, so the notion of Ubuntu, central to many traditional southern African communities, encapsulates a sense of communitarianism. *Umuntu ngumuntu ngabantu*, a person is a person through other persons, often interpreted as *I am because you are*, speaks to the African philosophical approach of interconnectedness of humans in society.

While Ubuntu is described as being of southern African origin,^29^ others argue that the notion is widely prevalent in other parts of Africa, but is referred to using different terminology.^30–32^ “Analytically speaking, ‘Ubuntu’ is a term used to describe the quality or essence of being a person among many sub-Saharan tribes of the Bantu language family.”^33^ Communitarianism further affirms that individuals are socially embedded, and that personhood is woven into the community with communal good taking priority over individual priorities.^34^ Eze, however, argues that “identity or subjectivity of the individual and the community is mutually constitutive and hence none is supreme.”^33^ The extent to which this approach is practiced varies from urban to rural communities and among younger and older individuals in different parts of the continent.

The concept of relative solidarity described by Ogunrin et al. reflects this nuanced application of the principles of Ubuntu.^32^ There has been a generational shift demonstrated between the older African generation who subscribe more commonly to the above communal approach to ethics and research in general, while the youth appear to be more enmeshed with Western Philosophical thought that places a higher value on personal autonomy and self-interest.^31,32^ The relative solidarity model allows for the amalgamation of these contrasting worldviews. This model works in the context of responsive communitarianism, as opposed to classical or authoritarian communitarianism. The main thesis of responsive communitarianism is that people face a conflict of two major sources of normativity: that of the common good and that of autonomy and rights with neither principle taking preference.^30^

While Ubuntu intersects closely with CE in southern Africa, the value of reciprocal relationships between researchers and communities has also been emphasized in New Zealand, where drawing on indigenous knowledge and Maori protocols and practices has proven invaluable to those involved in biobank research. This bridging of the communication divide has allowed for a greater practice of co-production and reciprocity of knowledge.^35,36^

While CE grounded in relative solidarity is important in all types of research in African settings, it has particular relevance in the field of genomic biobanking because these fields of research have medical implications for individuals, families, and communities. Genetic research involves the study of DNA, the basic unit of heredity, while genomic research involves the study of the entire human genetic code, known as the genome. This includes the analysis of human material and requires the recruitment of individuals.^37^

Biobanking involves the collection and storage of biological material, often in the form of blood, saliva, and urine, as well as demographic and health-related donor information. Biospecimens and data are anonymized and stored for future use. The success of genomic biobanking lies in the generation of big data and sharing of biospecimens and data in a global research community. This is well demonstrated in the success of the UK Biobank, which has a cohort of over 500,000 samples that are being successfully used to study the epigenetics and predictive lifestyle factors of cardiovascular health events, in addition to a variety of other outcomes.^38,39^

Genomic biobanking, which focuses on the storage of genetic material, has gained a foothold in the research culture of first world countries, with biobanks established in the 1990s in the USA, Europe, Ireland, Estonia, and the UK.^40,41^ While this is a relatively novel concept in Upper Middle Income countries (UMICs), it is well developed, with China first developing a biobank in 2006 and the China National Genebank in Shenzhen, established in 2011, being one of the largest in the world.^42,43^ Most Lower Middle Income countries (LMICs) surveyed, however, have suboptimal infrastructure and regulatory frameworks related to biobanking.^43^

Biodiversity is one of Africa's richest assets. It is therefore unsurprising that African genetic material is coveted by scientists internationally. While genetic and other biological material from communities are partially protected by Material Transfer Agreements, biopiracy (the act of directly or indirectly taking undue advantage of research participants and communities in global health research) has a long and contentious history in Africa. This has occurred as recently as the West African Ebola outbreak from 2014 to 2016 when thousands of biological specimens left the continent without consent.^14,44,45^ While multinationals and foreign academics and institutions profit from Africa's biodiversity, there is minimal benefit sharing with local communities and minimal acknowledgment of local researchers and academics.^14,24,46^

The H3Africa project (Human Health and Heredity, Africa), funded jointly by the U.S. National Institutes of Health (NIH) and the Wellcome Trust, has begun the process of biobank development in Africa. While the aim is to enhance capacity development by encouraging African scientists to establish biorepositories in African countries, including South Africa, data and samples still leave the continent to collaborators abroad with broad consent and with minimal, if any, benefit sharing with local communities.^24,47–49^ Broad consent is controversial because participants consent to current and unknown future use of their samples, usually with approval of a Research Ethics Committee. However, this unknown future use may occur in a foreign country outside Africa. Some argue that unknown future use cannot be informed. While H3Africa regards capacity development as benefit sharing, local communities do not stand to benefit from the donation of their samples in more explicit ways.^48,50^ The use of broad consent has introduced a new discourse in health research, with commensurate ethical concerns, especially where broad consent is required to facilitate the continuing exodus of samples and data.^14,24,48,49^

While the large sample size and resultant predictive value of genomic analysis herald major advances in the assessment of community wide health, it also presents unique ethical challenges, in that the sampling may not be directly beneficial to the individual, and it may lead to the stigmatization of recognizable communities due to their association with major disease, such as with Tay Sachs and Ashkenazi Jews or Sickle Cell Anemia and Black African populations.^45,51,52^ The ethical concerns around genomic biobanking are exacerbated in the absence of authentic CE strategies.

## The State of CE, a Problem of Tokenism

While CE is an overwhelmingly positive component of research and is closely intertwined with a robust consent process, it is often seen in isolation and can be considered mere ritual, rubberstamping, window-dressing, or tokenism, riddled with power and technical imbalances, placation, dishonesty, poor representation, and a lack of legitimate control over the research processes by the community.^15,16,44^ Arnstein, in 1969, articulated the various levels of citizen participation, in the context of city planning, conceptualized as rungs on a ladder, ranging from nonparticipation (manipulation, therapy, and informing), degrees of tokenism (informing, consultation, and placation), to degrees of citizen power (partnership, delegated power, and citizen control).^53^

Tokenism is widely evident in CE in research settings where asymmetrical power relationships exist between researchers and participants and where perfunctory or symbolic efforts are made to tick boxes without authentic engagement and benefit sharing. Late involvement with communities, superficial engagement, or engagement as an afterthought is regarded as tokenistic CE.^54^ In South Africa, the most common type of CE involves the formation of Community Advisory Boards (CABs). While necessary as a component of CE, CABs are by no means sufficient. CE in general and CABs, more specifically, are often criticized as being ancillary to research.^55^

Similarly, resources allocated to the development and management of CE and CABs tend to be limited and are often the first to be cut from study budgets when research priorities are considered. In keeping with resource limitations, it is important to be explicit about what remuneration, if any, can be expected by members of the CABs. It is standard practice in South Africa that CAB members are reimbursed for attendance at meetings. This has the potential to attract unemployed community members who perceive participation as a source of income. Occasionally, the lines between CE and reimbursement may become blurred. This was our experience during the production of our biobanking video, when CAB members participating in the video demanded payment as professional actors.^56^

Reddy et al. identified four themes as important in the establishment of CABs, including a definition of purpose, membership and representation, power and authority, sources of support, and independence.^57^ There are three recommended methods by which CABs may be constituted. There is purposeful selection, where the members are chosen from groups interested in the project. A more democratic process entails election of members, while a mixed model combines aspects of purposeful and democratic processes.^58^ While CABs are constituted to represent the values and concerns of their communities, appointment of individual members may be based on accessibility and convenience rather than communal nomination. This may result in a lack of representation of those with time, resource, and transport limitations, including the truly destitute and poor, while the lack of veto power over research suggestions and approaches may lead to them serving primarily as sounding boards.

Notwithstanding the limitations of CABs, they have been shown to serve as intermediaries between the research team and potential or actual research participants. They often educate community members clarifying understanding to prevent therapeutic misconception.^9,13,59,60^

Despite the importance of CE in general and in genomic biobanking in particular, there is a dearth of research on this topic in resource-poor settings.^32,61,62^ Neither is there a suitable CE model. Consequently, we set out to develop a model based on our own research experiences in CE over the past 15 years, a literature review and empirical research in this field in a South African setting over the past 4 years. This article describes the culmination of our project on the ethical, legal, and social issues related to genomic biobanking.

## Development of the TRUCE Model

The Centre for Medical Ethics and Law (CMEL), at the Faculty of Medicine and Health Sciences, Stellenbosch University, South Africa, was awarded an NIH research grant to explore the ethical, legal, and social issues involved in Genomic Biobanking and CE in 2015. This project was conducted in conjunction with a biobank established at the neighboring academic Tygerberg Hospital. After Research Ethics Committee (REC) approval for the project was granted, the CE process began with broad SE. Early conversations began with members of the Biobanking CAB and entrenched biobank staff, researchers, clinicians, REC members and biobank donors, research participants, and patients. The TRUCE model was developed based on insights we gathered, while implementing a number of empirical studies and CE activities. See Table 1 for highlights of these activities.

**Table 1.** Highlights of Empirical Research and Community Engagement Activities That Informed the TRUCE Model

CAB consultation	The Biobanking CAB and staff were consulted to elicit their views on CE. A significant finding in this empirical work was the emergence of different definitions of community. This has informed the development of the TRUCE model and is detailed in step 1 of the model^14,24^
Educational video created	An educational video was developed in conjunction with CAB members as well as other research stakeholders—nurses, scientists, and clinicians working within the biobank. After a series of meetings and discussions, a script was developed with input from all stakeholders. A Whatsapp group was created specifically for communication with community members who contributed to the script. Participants in the video comprised CAB members, biobank staff, and medical students. Professional actors were deliberately not used to ensure a more realistic portrayal of the biobank and its processes. This video has been widely distributed and is available on YouTube.^80^
Script developed with input from all stakeholders	
Pamphlets created	Multiple pamphlets discussing biobanking, genetics research, and genomics research were created by members of the CAB, community workers, scientists, and communication experts, in conjunction with the research team. Early engagement in the development of these tools was appreciated by community members. This is elaborated on in step 3 of the TRUCE Model. The text was written in lay language and at this stage, it was clear that there were no words for genes in local languages. For English-speaking participants, the understanding of genes was poor even in young participants who had received a high school education. There was a perception among participants that although biology was taught well at their schools, genetics teaching was suboptimal. This prompted an engagement with senior educationalists in the South African Department of Education, who in turn facilitated a workshop with 100 high school biology teachers in the Western Cape. During this workshop, comprising both interactive lectures and informal discussion, important insights were shared in an attempt to co-create a new genetics curriculum for high school learners. The co-production of knowledge is detailed in step 5 of the TRUCE Model. An activity book on genetics and genomics was developed with similar broad input and workshopped with high school Life Sciences learners. Feedback from this community of learners and teachers was used to improve and edit the books.
Content presented in lay language	
Engagement with Department of Education and facilitation of workshops	
Co-creation of new genetics curriculum	
Activity book developed	
Continued dissemination	Both the pamphlets and the video continue to be disseminated broadly, to every reachable stakeholder. Return of results of the project is discussed further in step 8 of the TRUCE Model.
Empirical research	Empirical research was conducted, comprising interviews with experts,^14,24^ research participants, CABs, and other stakeholder groups (^36^ and unpublished data).

CAB, community advisory board; CE, community engagement; TRUCE, Tygerberg Research by Ubuntu for Community Engagement Model.

**FIG. 1.**
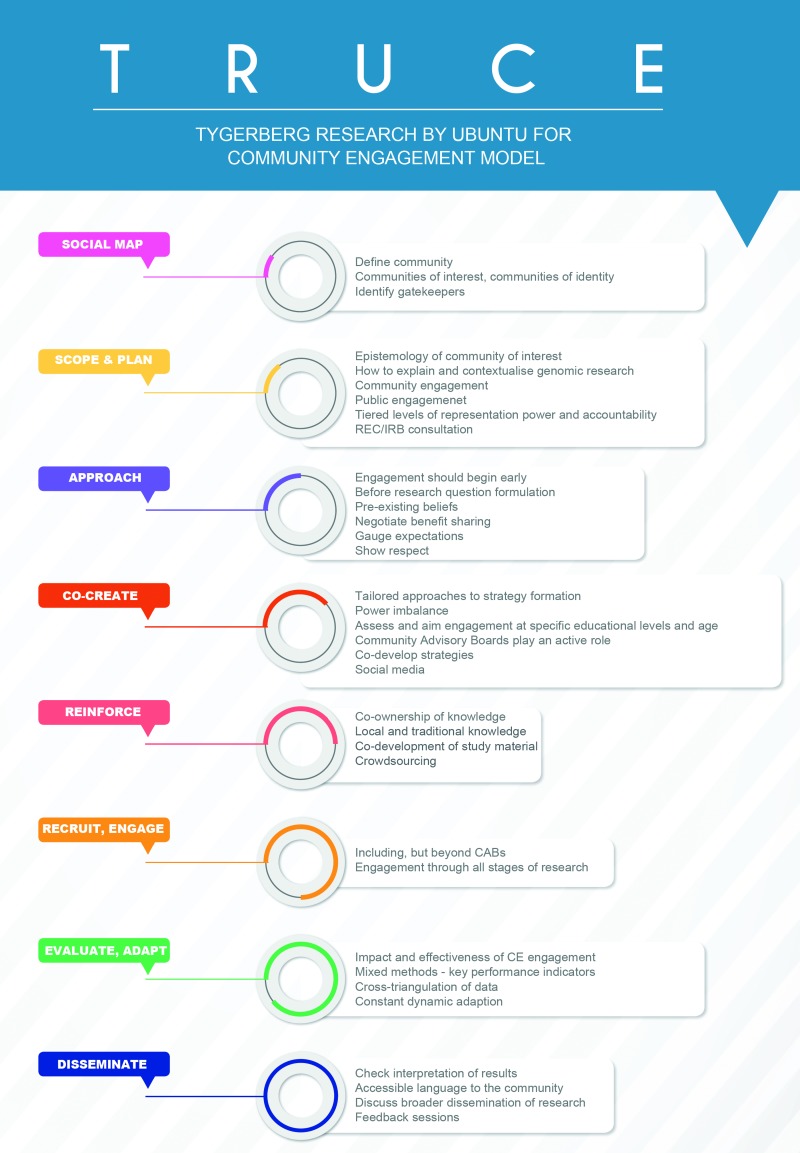
The TRUCE Model. TRUCE, Tygerberg Research by Ubuntu for Community Engagement Model. Color images are available online.

The TRUCE model, described below, is proposed as a model strategy for effective CE across all stages of a research project. While the contexts and limitations of each study will differ, following this model is likely to guide CE that is measurable, reliable, and relevant. Each step of the model is framed in the context of community consultation, which is a hallmark of Ubuntu. The TRUCE model consists of eight steps (Fig. 1):

(1) Use social mapping to identify potential communities.
(2) Set the scope of CE and plan accordingly.
(3) Approach communities early.
(4) Co-create strategies for CE.
(5) Reinforce co-ownership of knowledge production.
(6) Recruit and engage communities at each stage of research.
(7) Evaluate, reflect, and adapt CE strategies.
(8) Discuss and disseminate results.

### Use social mapping to identify potential communities

The concept of CE needs to begin with an assessment and appreciation of what a “community” is. MacQueen discusses the five defining characteristics of a community as follows: locus, sharing, joint action, social ties, and diversity.^63^ It is important to recognize that there are communities of interest and communities of identity, and that each person may be involved in a multitude of communities based on their age, gender interests, or circumstances.^64^ This is analogous to the difference in motivation and expectation of patient groups in comparison with public groups, with the former advocating out of personal experience and for quality of medical services, with the latter advocating out of public good and for equity of medical services.^5,65^ CE needs to involve a multitude of relevant forms of engagement, to ensure that this heterogeneity of community is accounted for.

There are multiple potential communities of interest that are specific to age, race, gender, religion, or other demographic qualifiers. Therefore, they should be approached in a multifactorial way, identifying whether you are dealing with a geographic or demographic community as opposed to a community of purpose.^63,66,67^ The latter often includes patients and potential participants. These communities may develop and evolve over time, and this should be accounted for. In an empirical research study of our Ethical, Legal and Social Issues of Genomic Biobanking project, participants defined their community as one due to geographical commons^47^ (unpublished data). People in the community were “those living within close geographical proximity.” Some had the perception that their community stretched further to those in the same church, and to their family and circle of friends, “regardless of location.”

This is in keeping with the concept of Ubuntu, where neighbors are viewed as members of the extended family with interdependent co-existence.^68^ According to Bongani Finca, “we don't live in isolation, we live in a community”.^29^ At the Tygerberg Biobank, the community of potential donors are the patients who live closest to the hospital and who fall into the catchment area of the health service.

Engaging a variety of stakeholders, including at the community level, helps to articulate a shared theory of change and drawing of social and network maps. Social mapping can provide valuable guidance for researchers to identify what strategies would work best in a given research context. Social mapping is a visual method of illustrating the relative location of households and the distribution of different types of people by specifiers such as gender; age; ownership of land; and literacy level, together with the social structure and institutions of an area. An analysis of these data will help define communities of relevance to the proposed research. In our empirical work, respondents described three different layers of SE: community level, peer level, and high level. Community-level engagement included potential participants, CABs, and field workers, and peer-level engagement included researchers, biobankers, and scientists, while high-level engagement included government officials, funders, and policy makers.^24^ Comments elicited from a peer-level stakeholder group was as follows: “When we export material from SA or from underdeveloped countries to the developed countries, we have no control over its eventual fate. Are we being used as experimental collection depots for the third world?”^14^ Although education of each stakeholder layer is important, education of the community layer is most challenging, due to the complexity of the research and educational levels of stakeholders in this layer.^24^ Consequently, the TRUCE Model focuses on CE at this level.

Often, senior members of the communities, such as chiefs, serve as gatekeepers, while others regarded as community leaders include religious leaders, teachers, influential people, celebrities, politicians, or council members such as the San Council in southern Africa.^1,20,69,70^ Approaching gatekeepers to communities must only be seen as an early step in the CE process. Individual members of the community retain the right to participate in the engagement process as they choose. With the San community in South Africa, where genetic and genomics research occur frequently, researchers may only approach the community after the San Council has agreed. Subsequently, the community discusses the research plan and votes about whether they should participate in the study or not. Each member of the community then provides consent individually. Research can then proceed in keeping with the San code of ethics.^62^ A judicious choice of methods for bringing people together, and supporting their debate and decisions, depends upon the drives and characteristics of those involved, particularly their degree of enthusiasm, experience, and motivation.^71^

### Scope of CE and planning

The scope of CE needs to be determined at the onset. This will depend on the type of research being conducted as well as demographics of the relevant communities.^72^

Once communities are defined, establishing the scope of CE will determine whether specific communities will be approached or whether a wider type of public engagement needs to occur. It is important to keep the scope of purpose and objectives in line with relevant legislation, regulations, and overarching aims of the research institution.^72^ Communication with RECs is critical at this stage. It may be necessary to seek approval from the REC to conduct formative research in the community ahead of the actual research project. In large projects, approval of the main research study may take several months. In this time, CE can start at a generic level, testing perceptions around important concepts planned for the larger study. In the case of genomic biobanking, testing the acceptability of broad consent may be relevant using hypothetical scenarios. Empirical research conducted with 200 participants at six clinical trial sites in South Africa indicated that almost half the sample did not support broad consent.^50^ Likewise, assessing understanding of DNA and genes may be relevant (Asian Bioethics Review + unpublished data).^47^ In other projects, like emergency trauma research, it may be necessary to assess perceptions on delayed consent before conducting the actual study.

How do we assess the degree of understanding or approach to systems of knowledge prevalent in the relevant community? Genomic biobanking involves particularly complex scientific concepts that many communities, even in developed world contexts, do not fully understand. Our empirical work with patients revealed that the concept of genetic inheritance is often best approached by creating a family tree and noting degrees of similarity between familial individuals as well as diseases prevalent in more than one member (unpublished data). It is also important to establish how each community approaches knowledge. While some communities are more influenced by the scientific method, others place more value in the opinions of elders and established cultural traditions.^35,59,73,74^

Implementation research considers a range of conceptual, ethical, and practical issues relevant to CE, while determining how the study will ensure adequate and equitable representation from underserved communities.^21^ Applying systems thinking methods and tools assists in understanding and analyzing complex system dynamics and relationships associated with specific interventions. Understanding the communities' concerns, cultural sensitivities, religious beliefs, barriers to participation, and potential misperceptions, as well as assessing the variations in resources, education levels, and purpose are relevant to the prospective scope of the CE process (unpublished data).^58^

In planning how to encourage research participation, purely altruistic appeals can be considered manipulative and can undermine the respect required in research endeavors. Solidarity and reciprocity are alternative prosocial appeals that have been shown to have more relevance and efficacy in encouraging participation in genomic research.^75^

### Approach communities early

Approaching community stakeholders must be undertaken in a way that is appreciative of their expertise as well as their availability.^2^ Ideally, engagement should start before formulation of the research question so that health needs and health priorities of the community can be established. Furthermore, early engagement allows researchers to assess community assets in terms of knowledge, skills, and resources. This baseline assessment allows the team to augment existing resources. Participants in one of our studies described genes as ‘something in the blood’ associated with inheritance, but also indicated that they “don't know where they are.”^50^ This guided the development of educational pamphlets, the educational video, and future interviews. Early engagement also informs the research team of pre-existing cultural beliefs. A CAB member indicated, “Some people would perhaps say ‘No I can't, what are my ancestors going to say’ so they have to go and ask the elders first if they may do it.” Another CAB member indicated that, “You find native people like me, isiXhosa people, who find blood to be very sacred. If you have something of your own being in the possession of someone else, there's a danger involved, you see.” Unmasking these cultural beliefs occurred in our early conversations with potential participants.

Respect is an important principle in CE and early CE is a sign of such respect. The San community of South Africa has a specific code of ethics for research, which needs to be respected by all potential researchers.^70^ This level of respect should be extended to all members of relevant communities. Building a relationship with a community must be a primary aim in and of itself, with each study building on the work done by previous studies. The researcher, as a stranger to the community, needs to allow and facilitate the community getting to know them and becoming comfortable with their presence. This can be aided by advocating for the confirmation of the researcher's integrity as well as the integrity of their research institutions before eliciting participation from community members. It is important to be explicit about the intended and actual use of data, as communities in resource-restricted contexts are often wary of commercialization and exploitation of their data. If there is any prospect of commercialization, this must be made explicit.^62,76^ Communities must be empowered to negotiate benefit sharing early in the process of engagement. This is a requirement in the San Code of Ethics and applies to research on the San people in Southern Africa, but is also a general research ethics obligation based on the principle of justice. Those who bear the burdens of research should also benefit. Apart from providing assurance around privacy and the limits of confidentiality in genomics research, participants must be made aware of their right to withdraw consent for storage and use of their data and specimens at any time. Researchers must be trained specifically in communicating respectfully, resolving conflict with maturity, and responding to concerns relating to local contexts, and embedded social relationships. Ubuntu supports an approach toward a community of reciprocal relationships, considered *keystone to socially accountable medical practice* and has been described by participants as the foundational concept supporting a positive interaction between the reciprocal relationships and the social determinants of a community's health.^27^ Transparency is an important concept in early engagement. It is important to initiate an open and transparent discussion of what products and services will be made available to study participants to ameliorate the potential for participants to decide to participate in research based on inaccurate expectations of ancillary care, post-research care, and therapeutic misconception.^2^ Since monetary incentives have not been shown to increase recruitment, this needs to be discussed in the context of existing guidelines.^61^ Early engagement is a sign of respect as the community is then aware that they are not being involved as an afterthought. A culture and relationship of trust can be built and maintained by demonstrating the value the research team sees in members of the community, beyond their involvement in any specific study. The consent document itself needs to be created with the direct, and complete involvement of the community, often by way of their representatives, both on and off relevant CABs. The value in this approach is that the concepts, expectations, approaches, interventions, and prospective value or risks of the research can be fully understood by potential participants. This process goes beyond simply translating the language, as certain concepts in genomics and biobanking cannot be directly translated to African languages. These terms may be contextually explained allegorically to optimize understanding.^35,69^ Discussions around return of results is part of benefit-sharing. Where genetic and genomic testing are part of a project, return of results or lack thereof is critical. Prior studies have shown that individuals are more likely to enroll in large genomic studies and biobanks when feedback on Individual Research Results (IRRs) was offered, although not everyone is comfortable in receiving every type of result.^77^ Many participants appreciate the opportunity to set preferences, or filters, with regard to the types of IRRs. The potential benefits may include screening for disease, as well as forming reproductive decisions. Potential negatives include anxiety, confidentiality being problematic, career or insurance repercussions, and the risks regarding the penetrance of certain disease, not being well understood. When data or samples are stored in an anonymous format, the challenges with return of results must be explained. Communities must not be made to feel coerced into involvement, and it must be made very clear that their choice to participate, or, perhaps more importantly, not to participate, will in no way influence any aspect of their ordinary medical care.^14,24,47^ Problems may arise in terms of how consensus can be reached in a community with various cultural and hierarchical views. This process requires mutual understanding of the foundational aspects of research designs, taking into account the vastly differing epistemological bases of the community and research team.^76^ Researchers need to use various methods in advancing available evidence to achieve this mutual understanding. These methods may include workshops, poster campaigns, and/or social media, emphasizing the provision of regular updates to the community. This consensus is particularly important when creating consent documents. It is clear that early engagement enhances the consent process with mutual benefit to researchers and participants.

### Co-create strategies for CE

The strategy used to approach a specific group or community must be contextualized. In traditional African communities, approaching the chief and getting his consent, approval, and aid are beneficial. Engagement strategies must be tailored to the educational level of different communities. When we discovered that basic knowledge around genes and genetics was suboptimal, we started to engage with the Department of Education and biology teachers in the Western Cape. High school students can be approached through teachers, the School Governing board, and parents or caregivers. The youth can be best approached through social media, given their ubiquitous involvement in this digital space.^62,78–80^

Although CABs may play a role in the recruitment, enrolment, and follow-up participants, communities must be engaged in a manner that respects how they wish to be approached.

Where required, training and technical support for community members will be necessary to avoid a power imbalance that may render the CE process merely consultative, which, if not accounted for, may engender discontent and distrust in the community. Likewise, training of research staff to develop the necessary skills to engage effectively with communities is a critical pre-requisite in CE. Communication as well as negotiation skills and conflict resolution are central to building social capital.

### Reinforce the co-ownership of knowledge production

Knowledge co-production and ownership can be understood as an interactive and dynamic endeavor of multiple actors where conventional epistemological realms and roles of different actors are blurred.^80^ This draws attention to the dynamic changes that occur in understandings, norms, beliefs, and practices, where knowledge co-production serves as a source of learning.^81^ The co-ownership of knowledge that flows from the research should be emphasized as scientists and academics often take a disproportionate share of the credit for research outcomes. Co-ownership brings together local and traditional knowledge with scientific knowledge and authentically acknowledges the role played by communities in knowledge production. This is important to flatten hierarchies and reduce imbalances in power and privilege that are a hallmark of researcher-participant relationships. In our Genomic Biobanking project, various stakeholders, including CAB members, provided input into the development of educational pamphlets and the video script from the start. Some members of stakeholder groups participated in the videos as “actors.”^82^ The actual engagement with the community, relevant metrics for success, opportunities for engagement, and adjustment of aspects of the study must be mutually decided upon through rigorous debate and involvement with relevant CE representatives. The co-creation and democratization of knowledge involve incorporating all relevant stakeholders, including patients, doctors, researchers, and policy makers, in all phases of a research project from research design, and proposal appraisal, to patient recruitment, through to the interpretation of results and subsequent implementation of research outcomes.^81^ This process, aided by relevant crowd sourcing, allows for knowledge and services to be better adjusted to the needs and preferences of involved communities. Crowdsourcing involving outsourcing problems and tasks to a crowd, often through the internet and relevant technology, is particularly useful, cost effective, and rapid in health research.^83^ This leverages the collective intellect of online communities for specific goals and has been shown to improve intervention development and evaluation, as well as enhancing the facilitation of communication.^84^ The empowerment of communities in research should be a primary aim, with each subsequent project building upon this. A focus on the creation of varied, but equal roles in groups and subgroups, which build upon the skills of the individual members of the community, is vital. These groups, once sufficiently knowledgeable over the research process, may in turn be empowered as research oversight committees, working alongside Institutional Health Research Ethics Committees (HRECs) to govern standards of research conducted in their communities.^44^

### Recruit and engage communities at each stage of research

While CABs can be constituted in research studies to assist in managing the expectations of participants, this should not be the only form of CE. CABs have many roles and clear messaging regarding the research processes; benefits to potential participants and the community, with an explanation and discussion of the commensurate risks, are important.^13,47,57^ The roles of a CAB include, *inter alia,* the protection of community interests, while advancing research goals. In protecting community interests, they may focus on stopping exploitation and ensuring community benefit, while providing substantive input (from inception to conclusion) and building capacity by way of a culture of human rights and empowerment of communities. Research goals can be advanced by providing bilateral information sharing, acting as a public relations buffer, and assisting with recruitment. There is a feeling that CABs should educate the community on the study as well as serve as gatekeepers to the community, assisting researchers in gaining access into the community, but not engaging in active recruitment to avoid creating a sense of conflict. While CABs should not be directly involved in recruitment, they play an invaluable role in facilitating the informed consent process, helping in the development of materials that explain the study to participants, and representing the participants' concerns to the researchers.^85^ A researcher indicated in one of our studies that “Consent forms should evolve in consultation with CABs so that the consent form can actually contain the information that would be really important from the patient and community perspective.” He went on to say that, “At the moment the consent form is the product of the researcher/research team and biobank perspectives…it contains literature from an ethics and legal perspective…it may not be important from the patient's perspective.”^14^

CABs ought to advocate for full disclosure of the risks and benefits of the study to their constituent communities. It is important that CABs maintain their independent advisory voice, ensuring that their input into the research process is fair and constructive, respects the scientific process, and is in the best *self-identified* interests of community members; in this way, it is worthwhile for the CABs to account not just to the research team, but to the community as well.^58^ CE is fundamentally about building relationships, and how this relationship is built will determine the successful implementation and sustainability of research projects, including genomic biobanking.^86^ Potential participants should be invited to voluntary information sessions on the concept of genomic biobanking in general, and the study, specifically as part of the screening process. In our empirical research, discussions regarding the family tree were useful to conceptualize genetics and inheritance, and information was supplemented with pamphlets and educational videos. It is worthwhile to use several tools and media platforms to engage the community on a continuous basis and in an interactive manner through community newspapers, posters, radio, and social media throughout the life cycle of the research project. Historically, the focus of engagement has been in the earlier stages of clinical trials, such as protocol explanation and recruitment, with significantly less engagement in the later stages involving the results and dissemination thereof. Future CE must be explicit in not perpetuating this mistake.^87^

### Evaluate, reflect, and adapt CE strategies

The impact and effectiveness of CE need to be assessed to establish legitimacy within the broader scientific community.^47^ Mixed methods analyses can be useful in evaluating the effectiveness of the CE approach, while the cross-triangulation of data may provide deeper information around the nature of relevant research relationships.^3,36^

Key performance indicators must be established at the outset, from thematic analysis, including systematic and specific indicators to be evaluated by way of continuous, dynamic, mixed methods. Key issues identified include a limited conceptualization of CE, poor quality of methods reporting, unclear context validity of studies, poor reporting of context and process, enormous variability in the way impact is reported, little formal evaluation of the quality of involvement, limited focus on negative impacts, and little robust measurement of impact.^88^ The GRIPP-2 checklist is an example of a way of addressing these.^10,89^ The GRIPP2-Long Form is suitable for studies where patient and public involvement (PPI) is the primary aim, while the GRIPP2-Short Form is suitable for studies where PPI is a secondary focus.^10^ Another example is Khodyakov's CE in Research Index, which covers each aspect of the research process, and whether the community was actively engaged, consulted, or did not participate.^90^ The CE strategies employed should be adapted dynamically within a study, as well as for each subsequent study. Realistic evaluation can assist in this adaptation of strategies. This form of evaluation aims to find out the contextual factors that make interventions effective, to understand why interventions work in some conditions, but not others.^4^ These evaluations are unique in their approach as they begin with a theory about how actions and mechanisms work in each particular context to create a desired outcome. This process is repeated in an iterative manner that aids in generating a hypothesis about what specific aspects of an intervention affect change, and specifically which groups may benefit. These results are fed back into the process to create a series of studies that each advance the next.^4,78,91^

### Discuss and disseminate results

Following review of the research by the research team, the analysis and dissemination of the results to the community and specific stakeholders is an important final step. This should follow relevant communication channels developed during the CE process using easily understandable language.^92^ Discussing the research results is important to check if the interpretation of the research team was correct. Involving the CAB or other community members who participated in the research process, in screening lay publications and in advising the research team, is important.^93^ These stakeholders could also help to facilitate understanding in the community by contributing to the feedback sessions. The research team should also, at this point, discuss the plans for broader dissemination of the research with the community, paying particular attention to accrediting the community for the role they have played in the research, and the way in which they would like to be acknowledged in subsequent publications. In acknowledging communities in research publications, it is important not to inadvertently offend or stigmatize them. For example, in research on the San community, the San Council has articulated a preference for use of San and Khoi separately, but not Khoisan (personal communication). Stigmatization of communities with HIV or TB can also occur, especially through publication in the media where specific geographical areas become associated with specific diseases.

## Limitations of Our Model

Our model is designed for a developing world, urban setting, and therefore the approaches may not be broadly generalizable to the developed world, or to rural settings. While we made a concerted effort to appraise all available literature on this topic from medical, allied health sciences, and social sciences, we did not conduct a systematic review or meta-analysis of data available as both had been done fairly recently. We did, however, consult with prominent researchers on this topic, and do not believe that the lack of a systematic review made a material difference on our approach. There still exists a dearth of literature examining biobanks in Africa specifically, and developing countries in general, which makes creating a model especially difficult. While the empirical work conducted for the ELSI Genomic Biobanking project contributed to the development of the TRUCE model, the broad principles implicit in the model can be extrapolated to other types of research. The model proposed in this study remains to be evaluated in a future project.

## Recommendations

Implementing the TRUCE CE Model is resource dependent. Consequently, the following recommendations are proposed. Making CE a funding requirement by including it in funding proposals and including it as a budget line item in grants is necessary where research is conducted among communities. Specific training in CE for research teams is essential. Furthermore, RECs/IRBs have a role to play in requiring a CE plan for every study that is community based, and scientific journals ought to require a paragraph on CE in publication of relevant research projects. Much of this can be ensured by moving CE from a guidance requirement to a regulatory requirement, emphasizing that it is a critical component of a robust consent process in research and that it ought to be embedded within research projects, where applicable. Communities in LMICs must not be left behind in the Fourth Industrial Revolution. Instead, we need to explore ways of harnessing digital technology to augment CE through wearables, mobile technology, access to data, and bandwidth. Such technology will make dynamic consent and durable engagement with tissue trusts and biobanks possible in low resource contexts.

## Conclusion

CE is critical in genomic biobanking in a resource-constrained setting where there is suboptimal understanding of genetics, genomics, and biobanking, yet it is an often neglected component of research. There is a significant gap between the theory of CE and application to conduct of research. There are also significant disparities between how CE is approached in resource-rich and resource-poor countries. The digital divide is expanding and technology must be harnessed to improve CE. It is hoped that the TRUCE model will facilitate the bridging of that gap by integrating the Ubuntu-derived concept of humanism and respect for dignity of research participants globally.
